# Supplementary figures and images for: Fibroblast growth factor receptor substrate 2 interactome mapping reveals novel candidate interactors associated with migration and invasion
Source: Cell Commun Signal. 2026 May 21;24:416. doi: 10.1186/s12964-026-02943-8 (PMC13393275; doi:10.1186/s12964-026-02943-8)

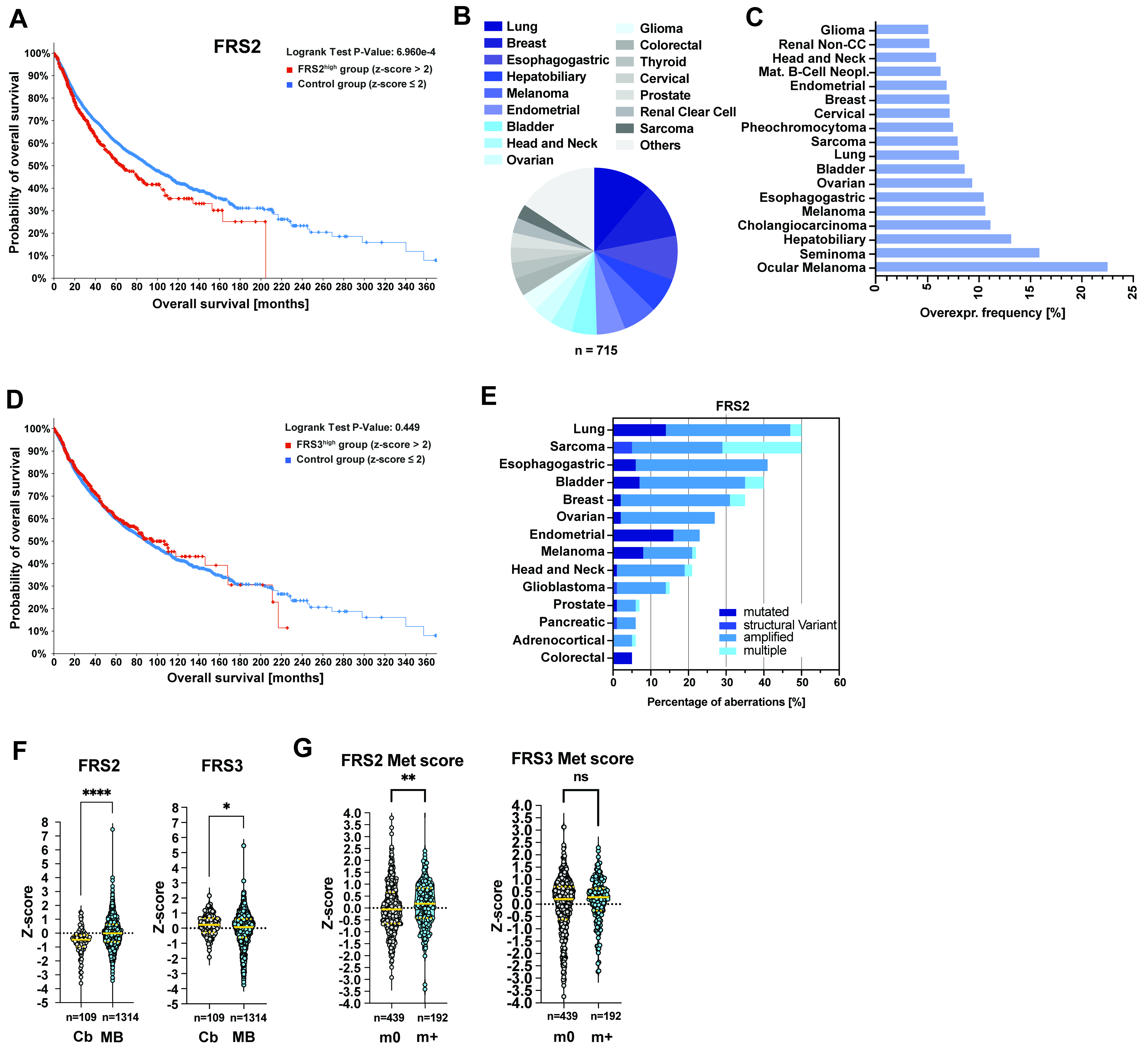

Supplement: Supplementary file 1 — Supplementary Material 1: Figure S1. A Kaplan–Meier survival probability curves for PanCancer patients with elevated (red) or unaltered (blue) FRS2 mRNA levels. B Absolute number of TCGA PanCancer Atlas patients harboring altered FRS3 mRNA levels, stratified by diagnosis. C Relative frequency of elevated FRS3 mRNA levels per tumor diagnosis. D Kaplan–Meier survival probability curves for PanCancer patients with elevated (red) or unaltered (blue) FRS3 mRNA levels. E Proportions of FRS2 genomic alteration types stratified by tumor diagnosis. F Comparison of FRS2 and FRS3 mRNA expression Z-scores in normal cerebellum (CB) versus medulloblastoma (MB) tumor samples from the GSE124814 dataset. Statistical significance was assessed by unpaired two-tailed Mann–Whitney test: ns p > 0.05, *p < 0.05, ***p < 0.0001. G FRS2 and FRS3 mRNA abundance in relation to metastatic status in the GSE124814 dataset (restricted to patients with available metastasis annotation). M0: no metastases detected; M+: metastases detected [file 12964_2026_2943_MOESM1_ESM.tif]

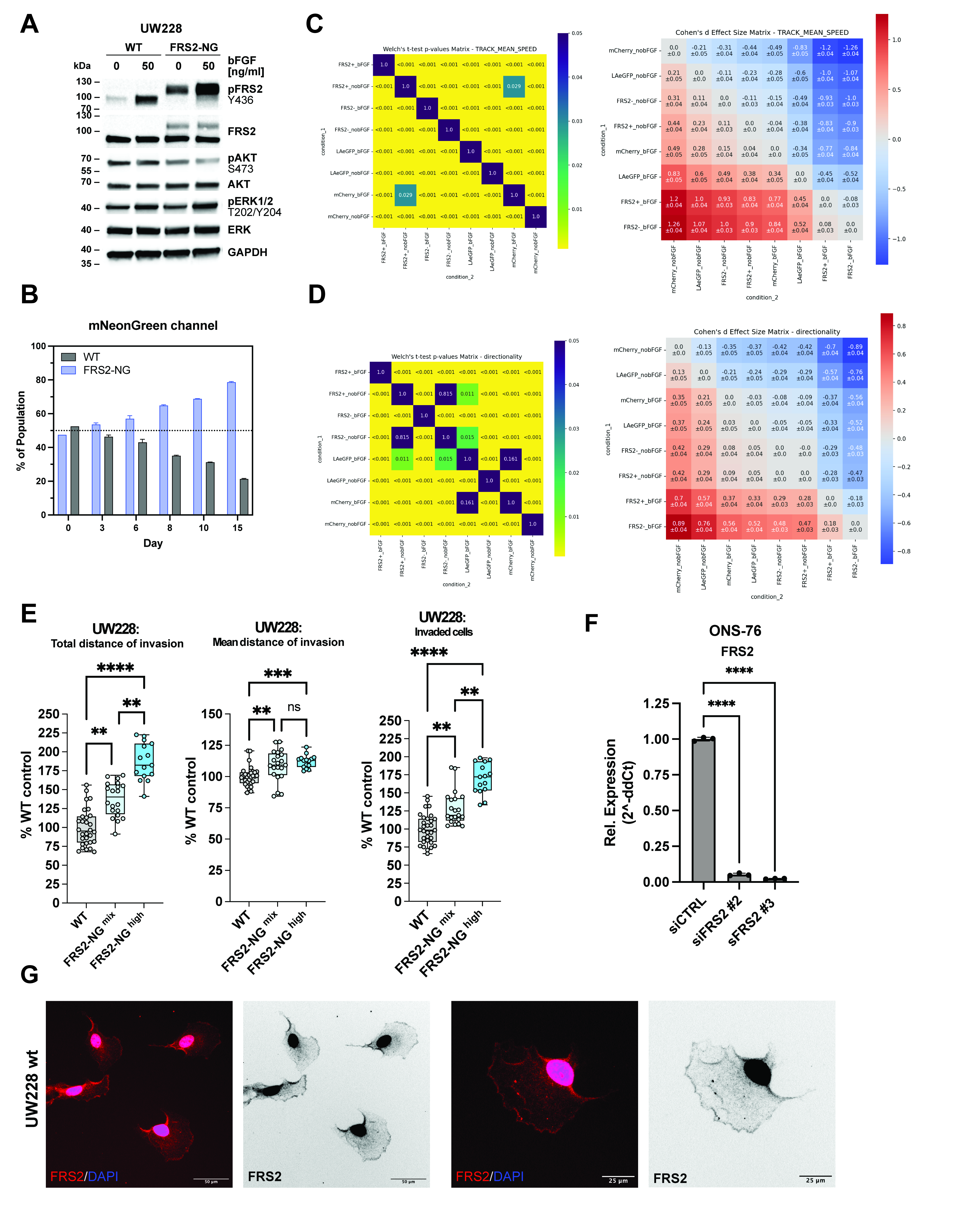

Supplement: Supplementary file 2 — Supplementary Material 2: Figure S2. A IB analysis comparing FRS2 phosphorylation and MAPK/ERK and AKT pathway activation in wild-type and FRS2-mNG-expressing UW228 cells. B Proliferation competition assay using co-cultured wild-type and UW228 FRS2-mNG/mCh-Nuc UW228 cells. The relative proportion of FRS2-mNG-positive cells was assessed by flow cytometry using the mNeonGreen channel. C, D Formal hypothesis testing and effect size matrices from live-cell tracking, comparing mean speed (C) and directionality (D) of UW228 cells overexpressing LA-EGFP or FRS2-mNG (FRS2−: mixed FRS2-mNG expression; FRS2+: high FRS2-mNG expression; LA-EGFP: control). E Spheroid invasion assay (SIA) comparing wild-type and FRS2-mNG-overexpressing UW228 cells in the presence of 100 ng/mL bFGF. Dots represent individual spheroids (each comprising > 2,500 cells) from technical replicates of n = 1 experiment. Statistical significance was assessed by one-way ANOVA with Kruskal–Wallis and multiple comparisons correction: *p ≤ 0.05, **p ≤ 0.01, ***p ≤ 0.001, ****p ≤ 0.0001. F Validation of FRS2 knockdown efficiency for siFRS2 #2 and siFRS2 #3 by RT-qPCR in wild-type ONS-76 cells. G Immunofluorescence analysis of endogenous FRS2 localization in wild-type UW228 cells [file 12964_2026_2943_MOESM2_ESM.tif]

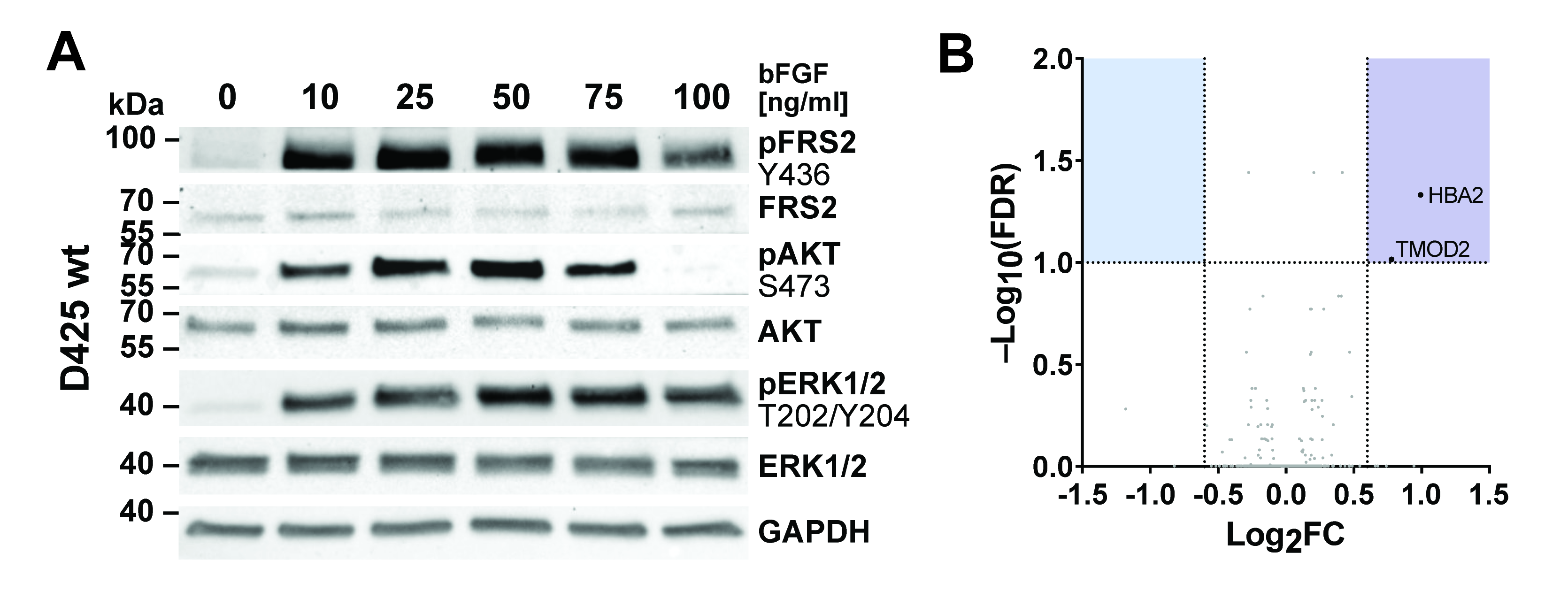

Supplement: Supplementary file 3 — Supplementary Material 3: Figure S3. A Determination of the optimal bFGF concentration for FGFR signaling activation in D425 cells by IB analysis following 5 min stimulation. B Total proteome changes in D425 cells stimulated with 50 ng/mL bFGF for 5 min. Gene names of significantly upregulated proteins (FDR ≤ 0.1, log₂FC ≥ 0.6) are labeled in the upper right quadrant [file 12964_2026_2943_MOESM3_ESM.tif]

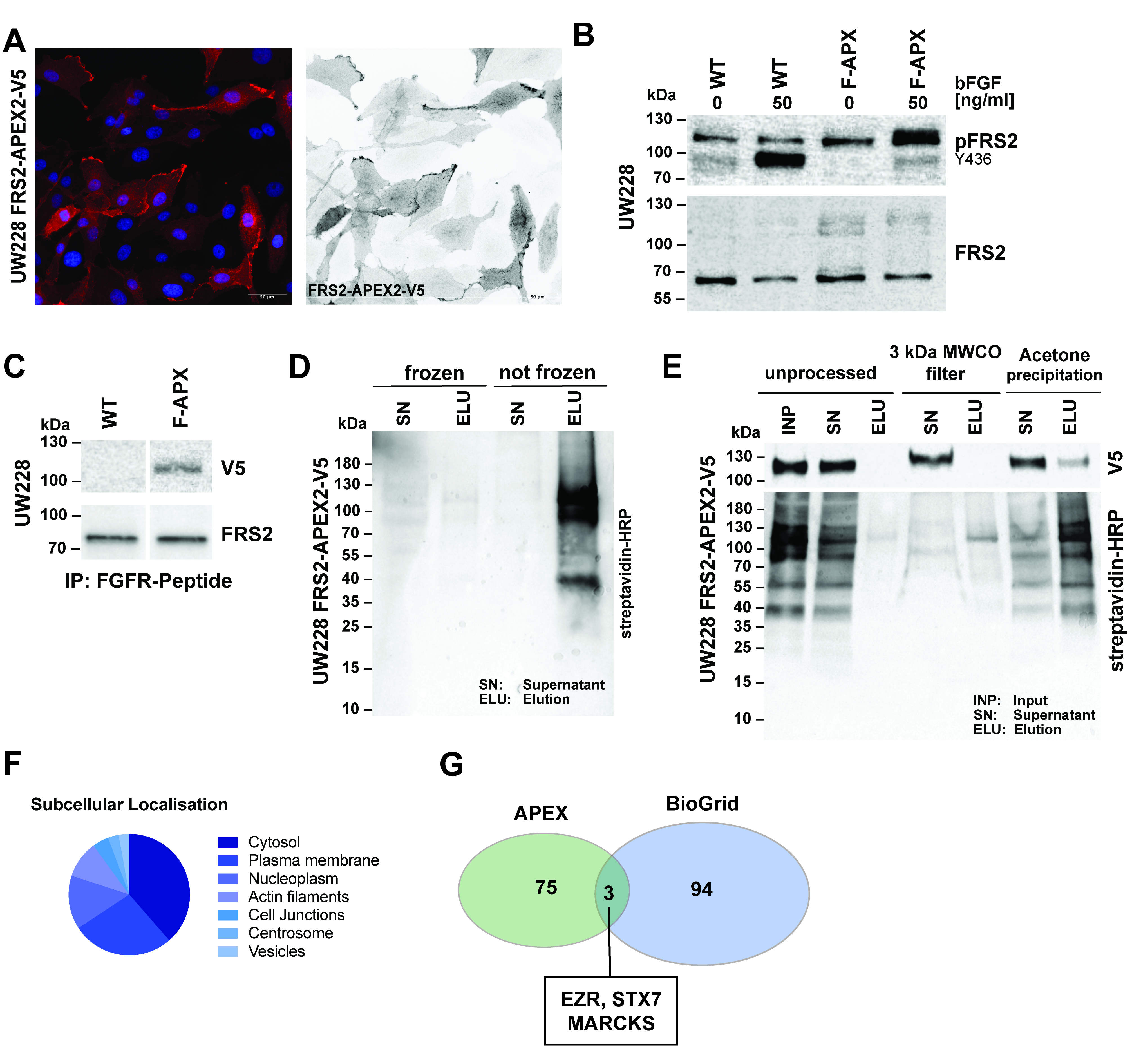

Supplement: Supplementary file 4 — Supplementary Material 4: Figure S4. A Subcellular localization of FRS2-APEX2-V5 in UW228 cells assessed by IF analysis. The transgene was detected using an anti-V5 antibody. B Comparative IB analysis of FRS2 phosphorylation in wild-type UW228 and UW228 FRS2-APEX2-V5 (F-APX) cells following bFGF stimulation (50 ng/mL, 5 min). C IB analysis of eluates from wild-type UW228 (WT) and UW228 F-APX pull-downs using an FGFR peptide as bait. D IB detection of biotinylated proteins using streptavidin-HRP, comparing samples with and without pellet snap-freezing prior to lysis. E Comparison of filtration versus acetone precipitation for removal of excess biotin prior to pull-down, and their respective effects on biotinylated protein recovery. F Subcellular localization distribution of bFGF-induced FRS2 proximal interactor candidates identified by APEX2-based proximity labeling. G Overlap between APEX2-labeled proteins identified in the FRS2 proximal proteome under serum-free and bFGF-stimulated conditions combined and previously reported FRS2 interactors catalogued in the BioGRID database [file 12964_2026_2943_MOESM4_ESM.tif]

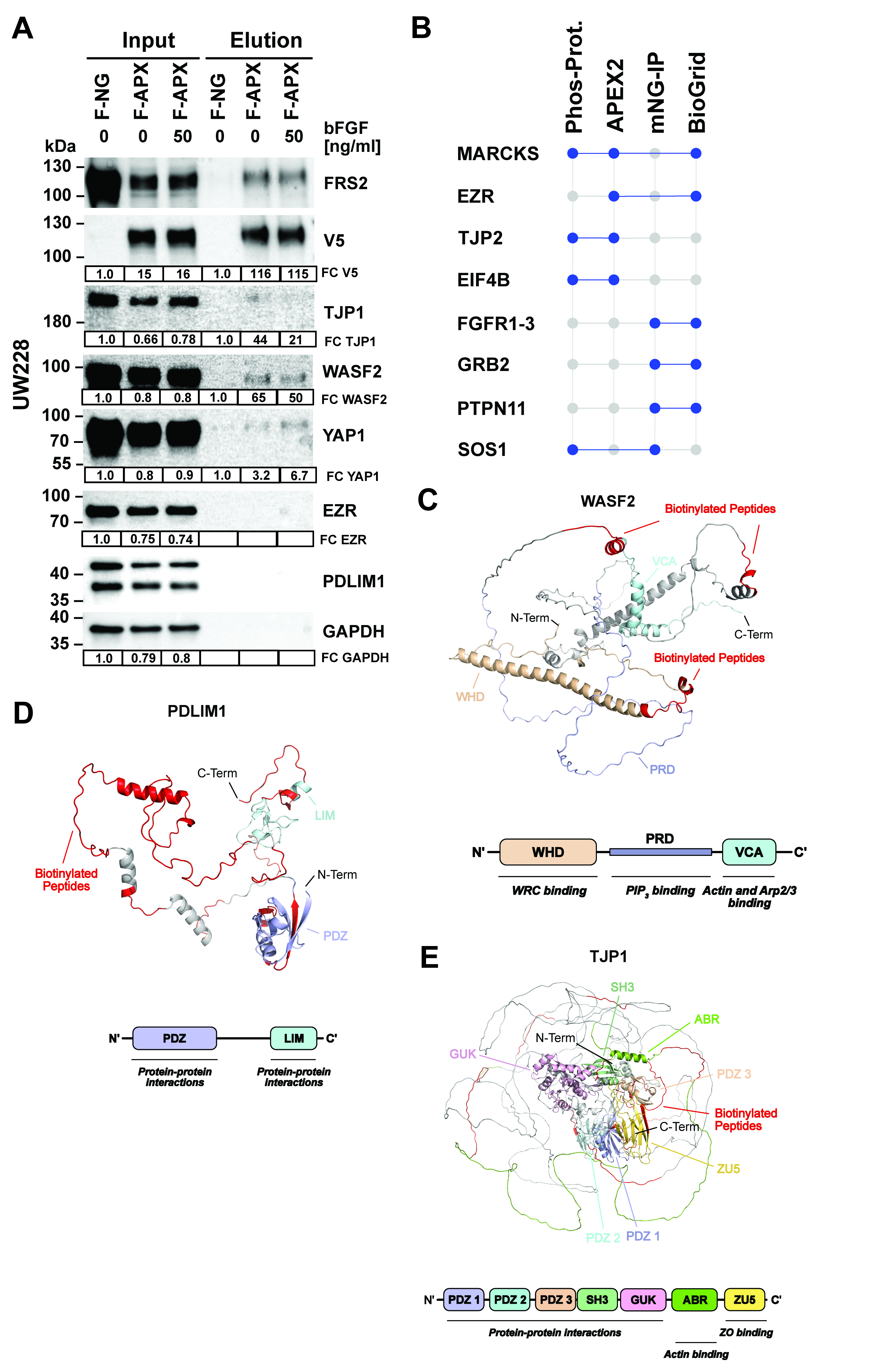

Supplement: Supplementary file 5 — Supplementary Material 5: Figure S5. A Validation of APEX2-labeled proteins TJP1, WASF2, YAP1, and EZR. Immunoblot analysis following streptavidin-HRP pulldown, probed with antibodies against the proteins indicated to the right of each panel. B Venn diagram visualizing shared hits across the following datasets: phosphoproteome analysis, APEX2 proximity labeling, co-immunoprecipitation, and predicted FRS2 interactors from the BioGRID database. C–E Biotinylated peptides identified by APEX2 proximity labeling (highlighted in red) mapped onto the predicted AlphaFold structures of WASF2 (C), PDLIM1 (D), and TJP1 (E). Schematic representations of the folded domain architecture are shown below each structure, with the general molecular functions of individual domains indicated [file 12964_2026_2943_MOESM5_ESM.tif]

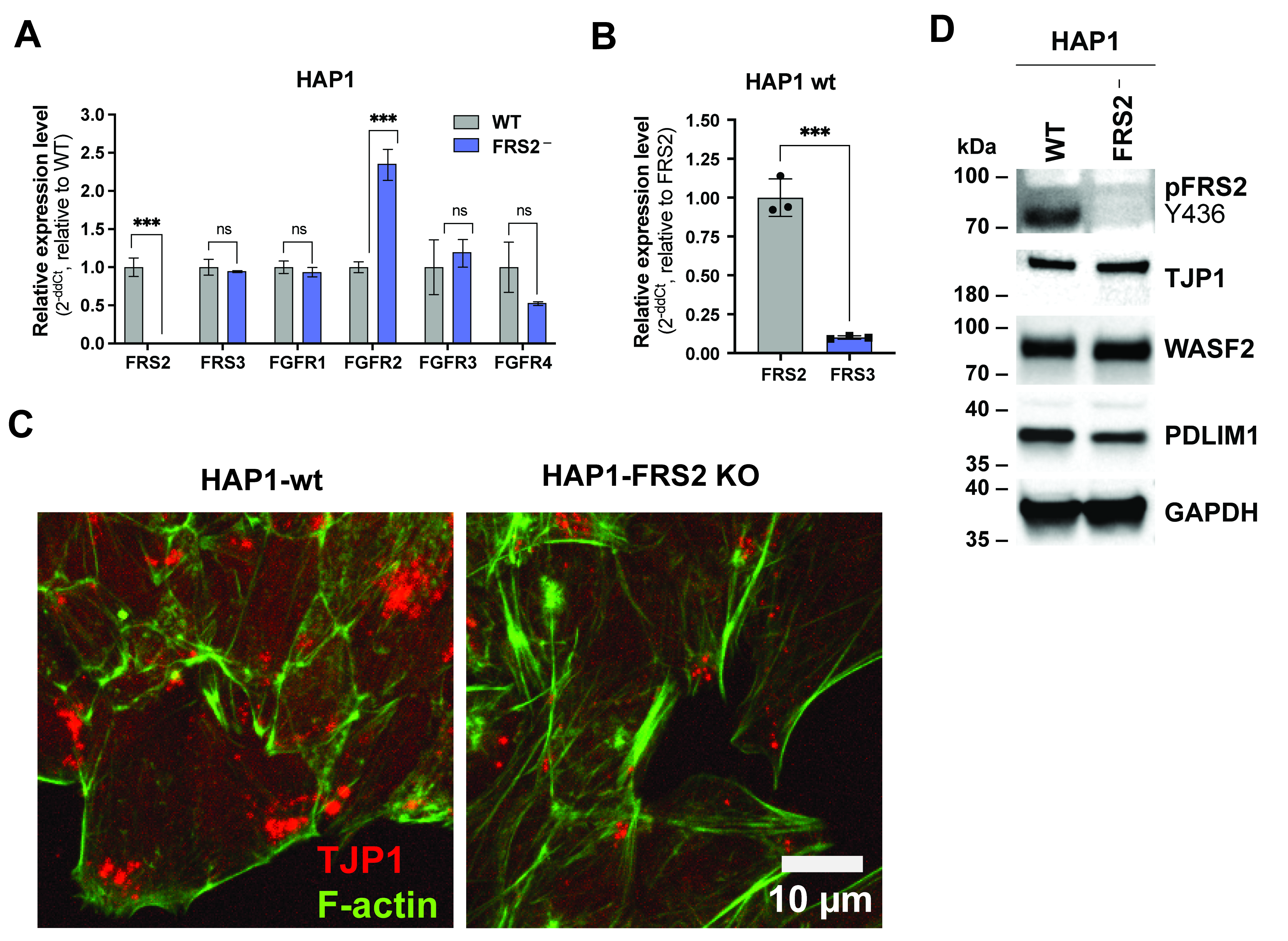

Supplement: Supplementary file 6 — Supplementary Material 6: Figure S6. A RT-qPCR analysis of FRS2, FRS3, and FGFR1–4 mRNA levels in HAP1 WT and HAP1 FRS2 KO cells. Statistical significance was assessed by unpaired two-tailed Student’s t-test: ns p > 0.05, ***p ≤ 0.001. B RT-qPCR analysis of FRS2 and FRS3 mRNA expression in HAP1 WT cells. C Magnified views of IFA images from Fig. 6A, showing HAP1 WT and HAP1 FRS2 KO cells stimulated with 50 ng/mL bFGF for 1 h. F-actin is shown in green (phalloidin) and TJP1 in red. D IB analysis of FRS2 interactor expression in HAP1 WT and HAP1 FRS2 KO cells [file 12964_2026_2943_MOESM6_ESM.tif]

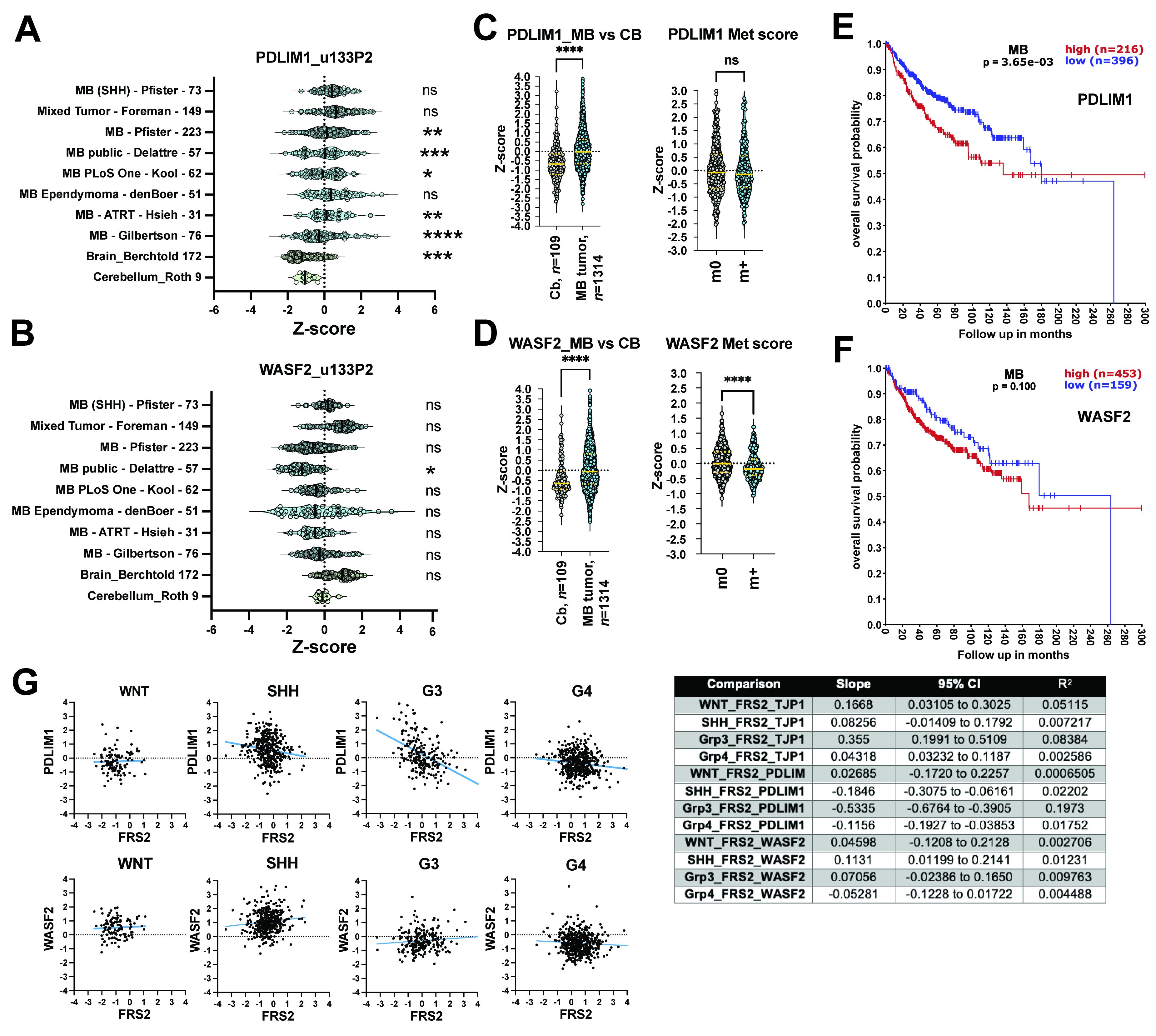

Supplement: Supplementary file 7 — Supplementary Material 7: Figure S7. A, B Z-scores of WASF2 (A) and PDLIM1 (B) mRNA expression across two control datasets (normal brain regions and cerebellum, shown in green) and six primary MB patient datasets (shown in blue), analyzed on the Affymetrix U133P2 platform. Significant differences relative to the healthy cerebellum control were assessed by one-way ANOVA with Kruskal–Wallis and Dunn’s multiple comparisons tests: ns p ≥ 0.05, *p ≤ 0.0332, **p ≤ 0.0021, ***p ≤ 0.0002, ****p ≤ 0.0001. C, D Left: Z-scores of PDLIM1 (C) and WASF2 (D) mRNA expression in cerebellar control (n = 109) and MB tumor samples (n = 1’314). Right: Z-scores of PDLIM1 and WASF2 expression in non-metastatic MB patients (M0) versus patients with tumor cells detectable in the cerebrospinal fluid (M+; GSE124814 dataset). Statistical significance was assessed by unpaired two-tailed Mann–Whitney test: ns p ≥ 0.05, ****p ≤ 0.0001. E, F Kaplan–Meier curves of overall survival in MB patients stratified by high (red) or low (blue) tumor expression of PDLIM1 (E) and WASF2 (F). G Left: Correlation plots of FRS2 mRNA expression Z-scores versus PDLIM1 or WASF2 across MB subgroups. Right: Corresponding slope statistics [file 12964_2026_2943_MOESM7_ESM.tif]
