# Supplementary material for: Fibroblast growth factor receptor substrate 2 interactome mapping reveals novel candidate interactors associated with migration and invasion
Source: Cell Commun Signal. 2026 May 21;24:416. doi: 10.1186/s12964-026-02943-8 (PMC13393275; doi:10.1186/s12964-026-02943-8)
Supplement: Supplementary file 18 — Supplementary Material 18. [file 12964_2026_2943_MOESM18_ESM.pdf]

Figure 2A

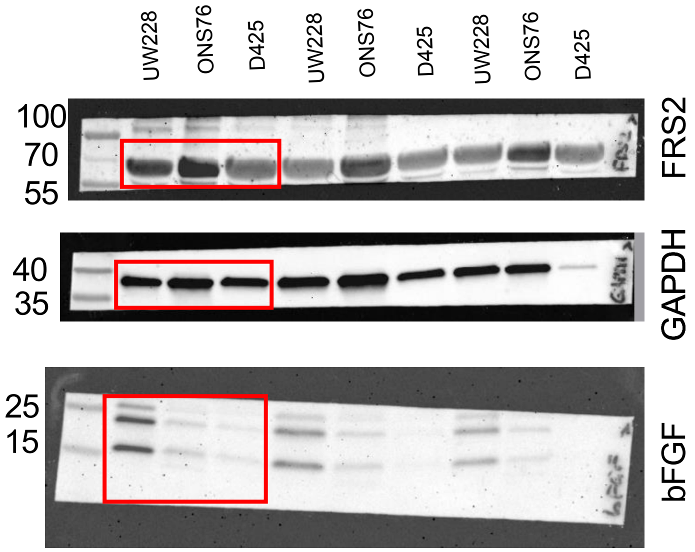
UW228
ONS76
D425
UW228
ONS76
D425
UW228
ONS76
D425
100
70
55
FRS2
40
35
GAPDH
25
15
bFGF

Figure 3A

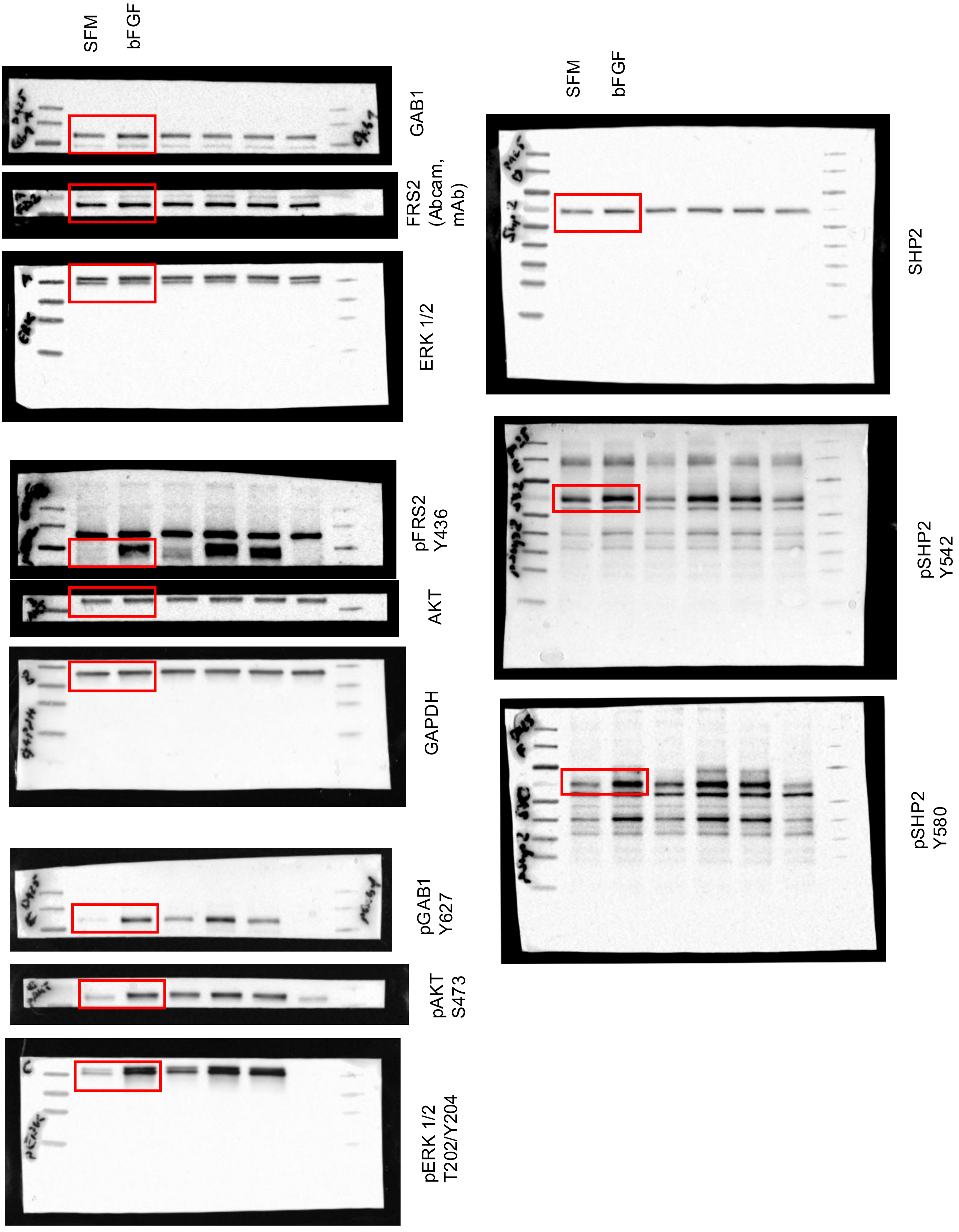
SFM
bFGF
GAB1
FRS2 (Abcam, mAb)
ERK 1/2
pFRS2 Y436
AKT
GAPDH
pGAB1 Y627
pAKT S473
pERK 1/2 T202/Y204
SFM
bFGF
SHP2
pSHP2 Y542
pSHP2 Y580

Figure 3E

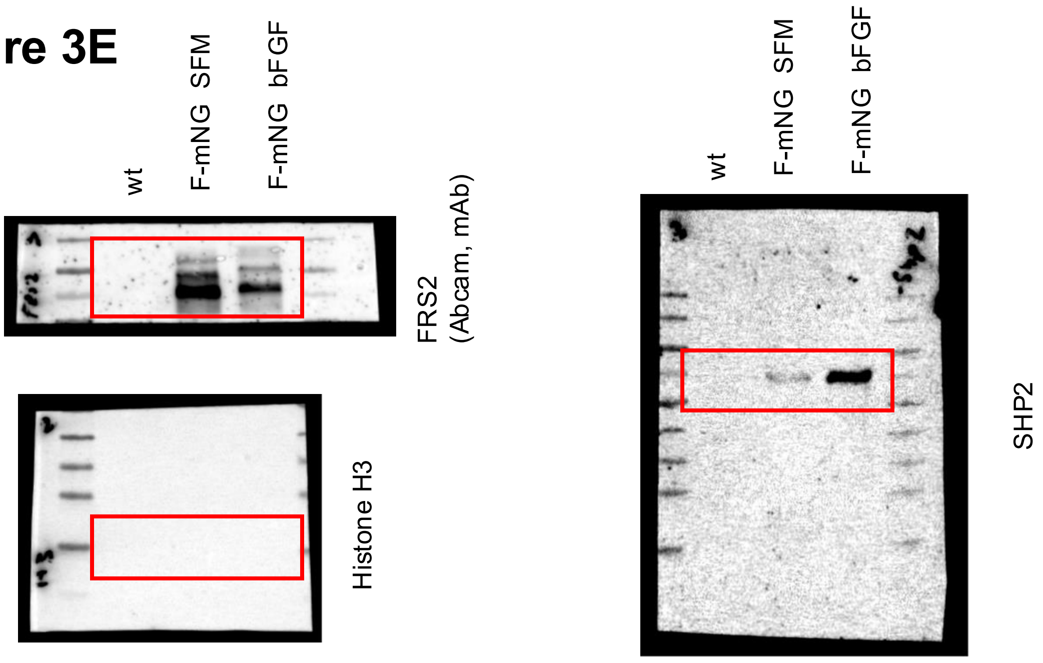

Figure 4B

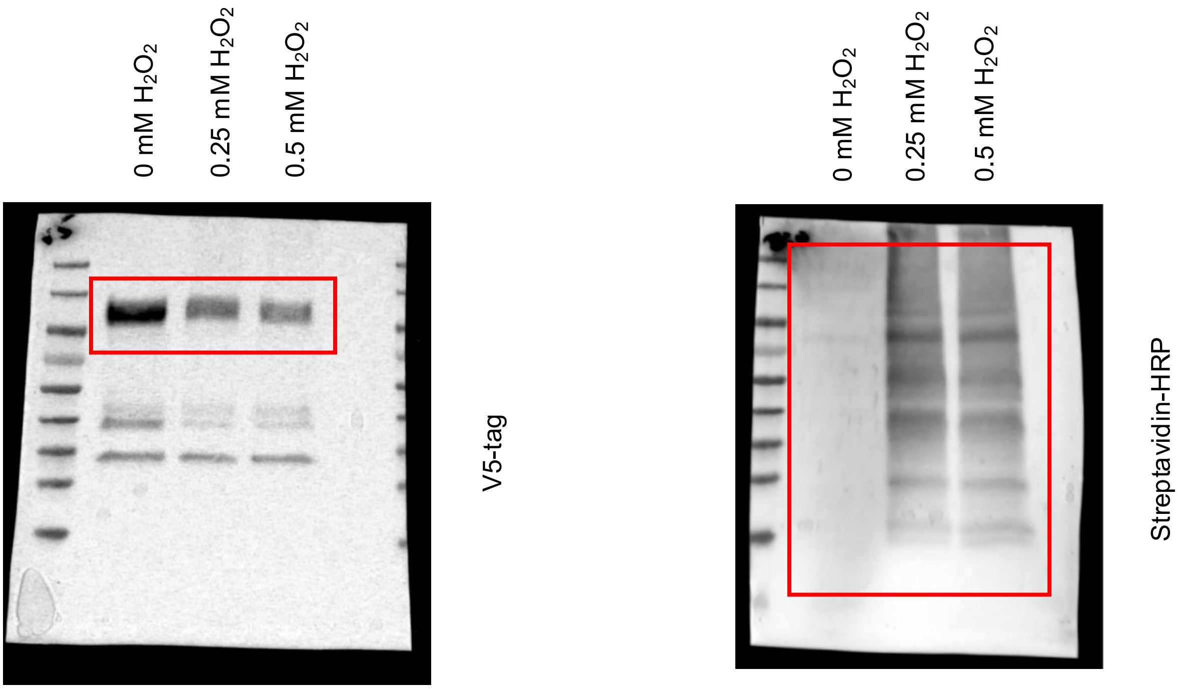

Figure 4C

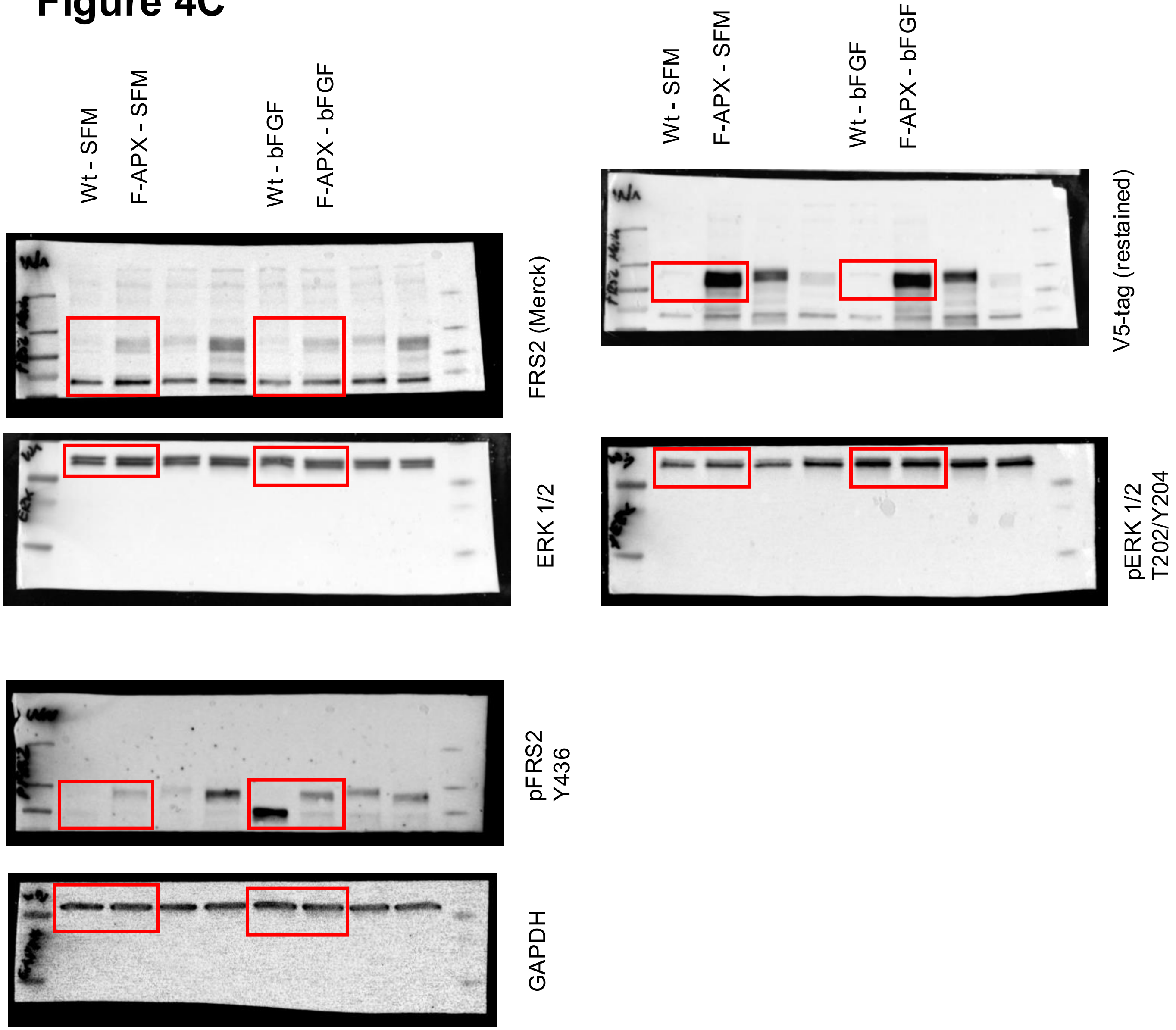

Wt - SFM
F-APX - SFM
Wt - bFGF
F-APX - bFGF
FRS2 (Merck)
ERK 1/2
pFRS2
Y436
GAPDH
Wt - SFM
F-APX - SFM
Wt - bFGF
F-APX - bFGF
V5-tag (restained)
pERK 1/2
T202/Y204

Figure 5B

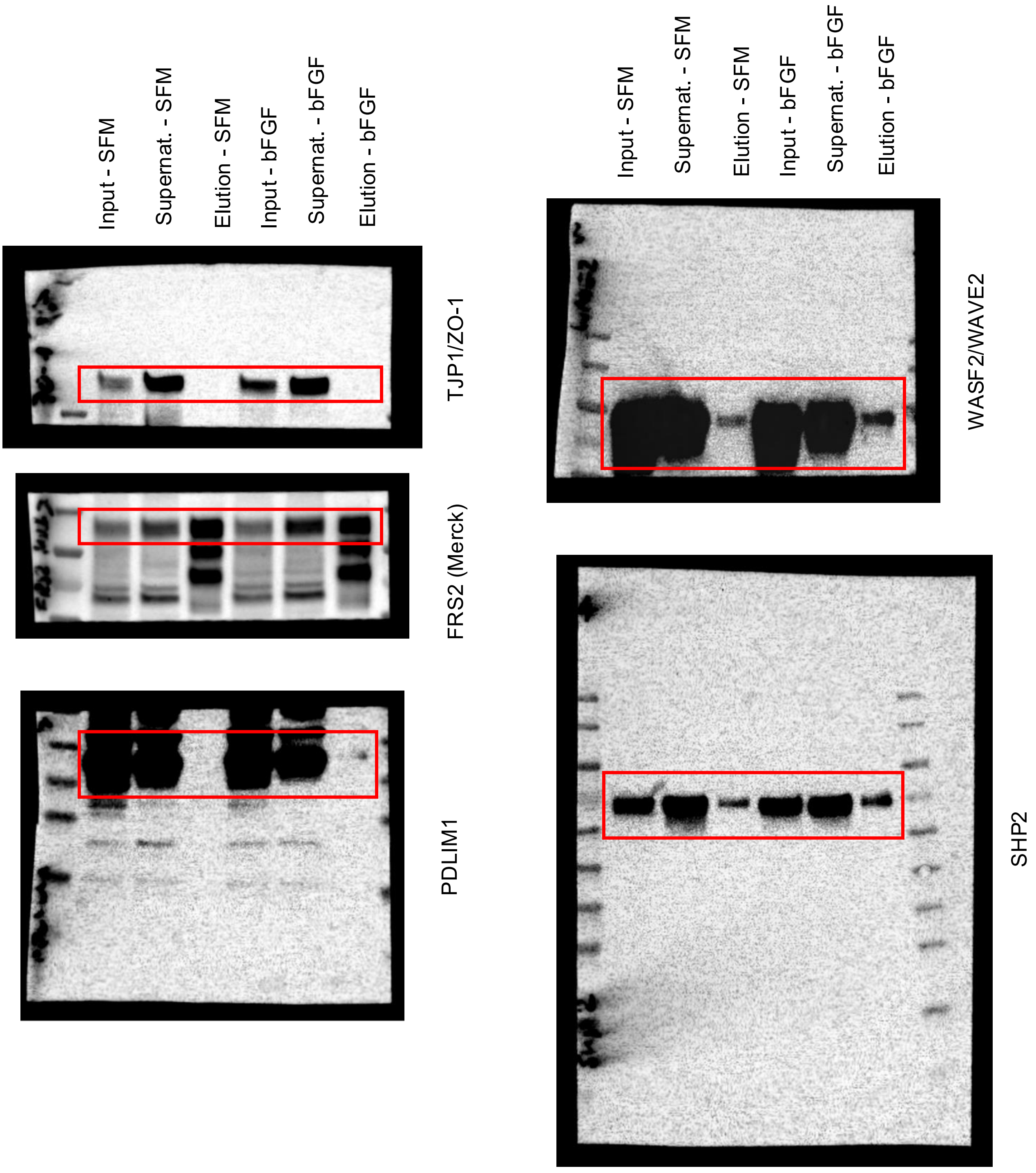
Input - SFM
Supernat. - SFM
Elution - SFM
Input - bFGF
Supernat. - bFGF
Elution - bFGF
TJP1/ZO-1
FRS2 (Merck)
PDLIM1
WASF2/WAVE2
SHP2

Figure S2A

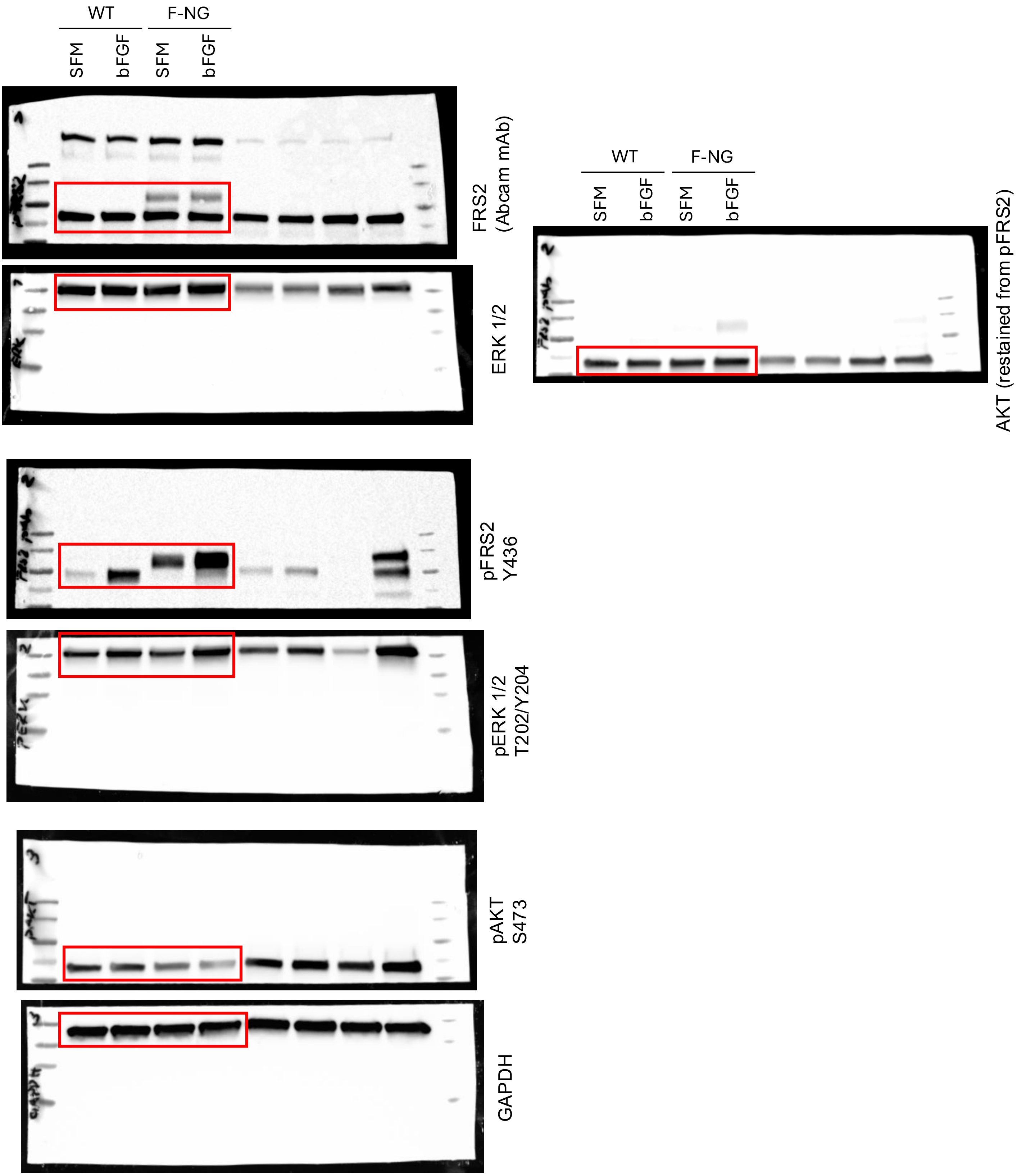

WT
F-NG
SFM
bFGF
SFM
bFGF
FRS2
(Abcam mAb)
ERK 1/2
pFRS2
Y436
pERK 1/2
T202/Y204
pAKT
S473
GAPDH
WT
F-NG
SFM
bFGF
SFM
bFGF
AKT (restained from pFRS2)

Figure S3A

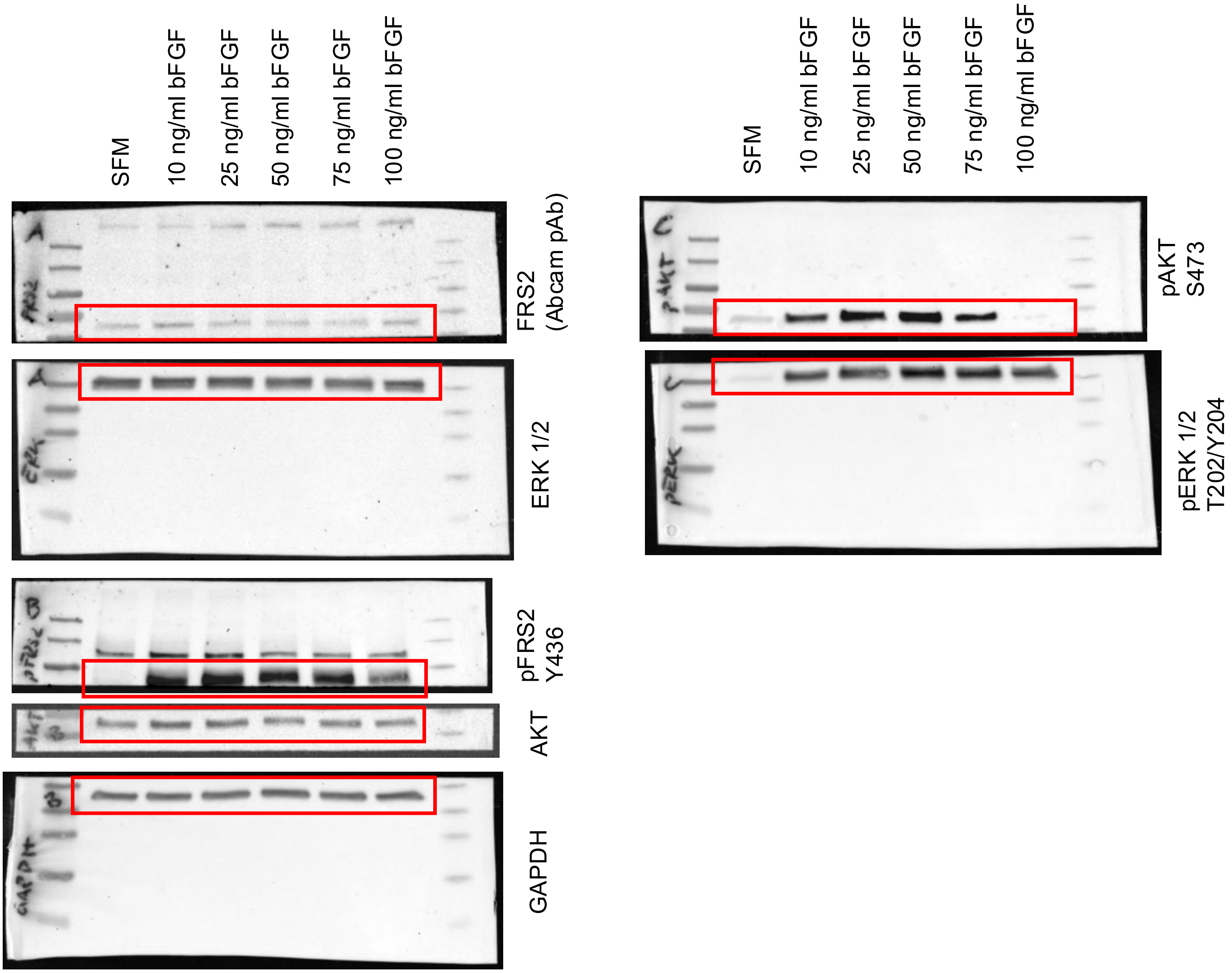
SFM
10 ng/ml bFGF
25 ng/ml bFGF
50 ng/ml bFGF
75 ng/ml bFGF
100 ng/ml bFGF
FRS2 (Abcam pAb)
ERK 1/2
pFRS2 Y436
AKT
GAPDH
pAKT S473
pERK 1/2 T202/Y204

Figure S4A

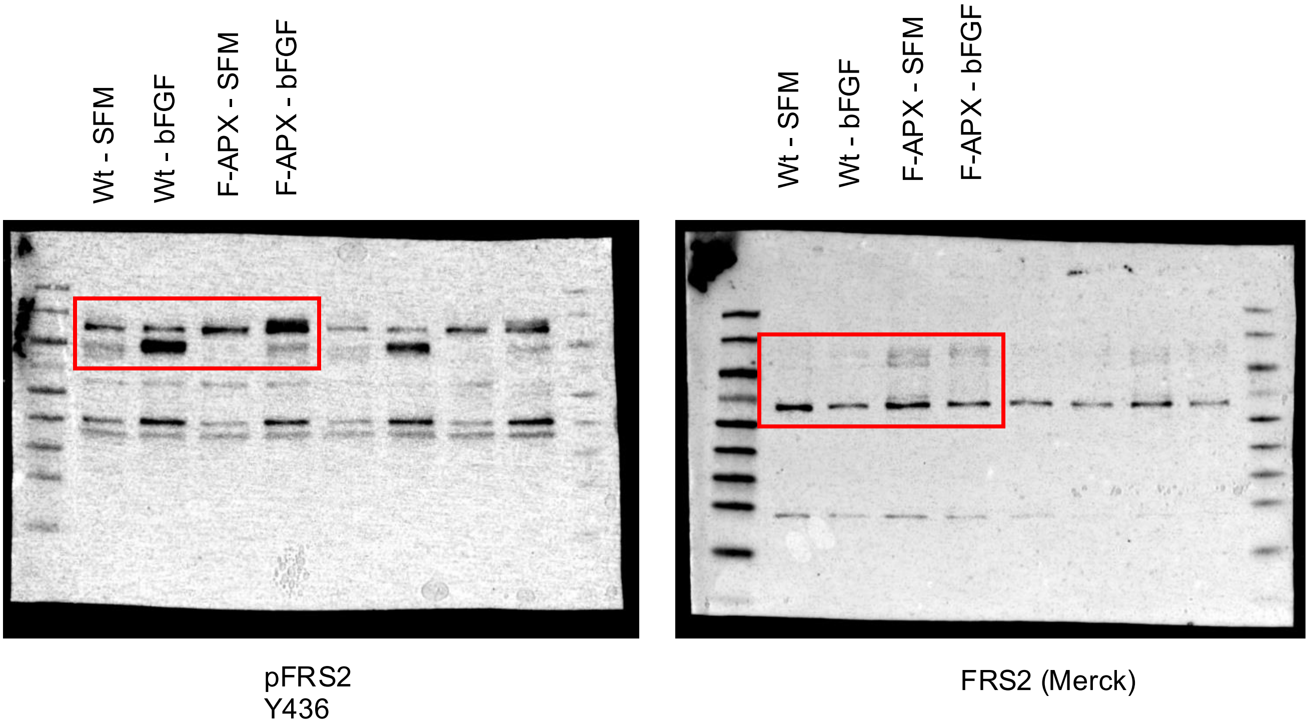

Figure S4C

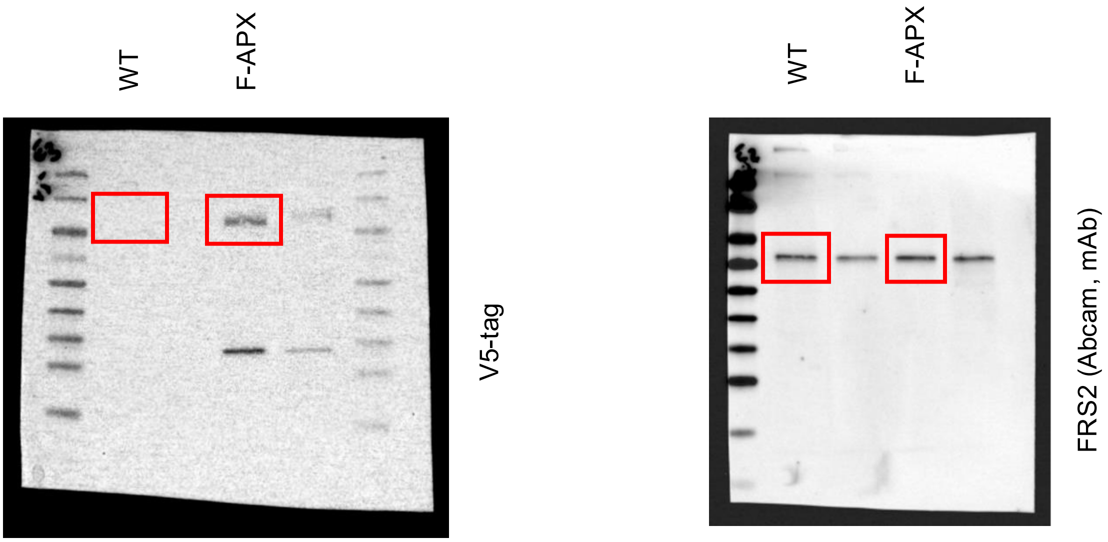

Figure S4D

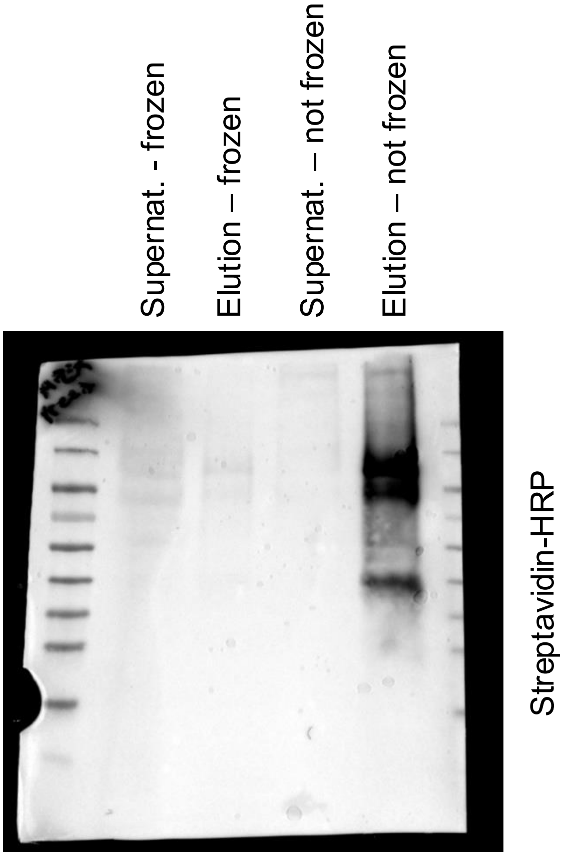

Figure S4E

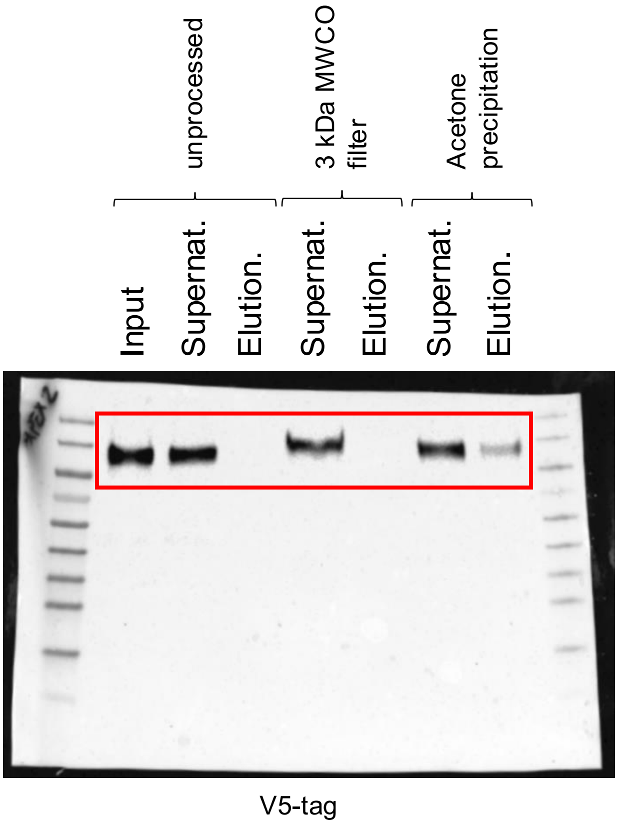

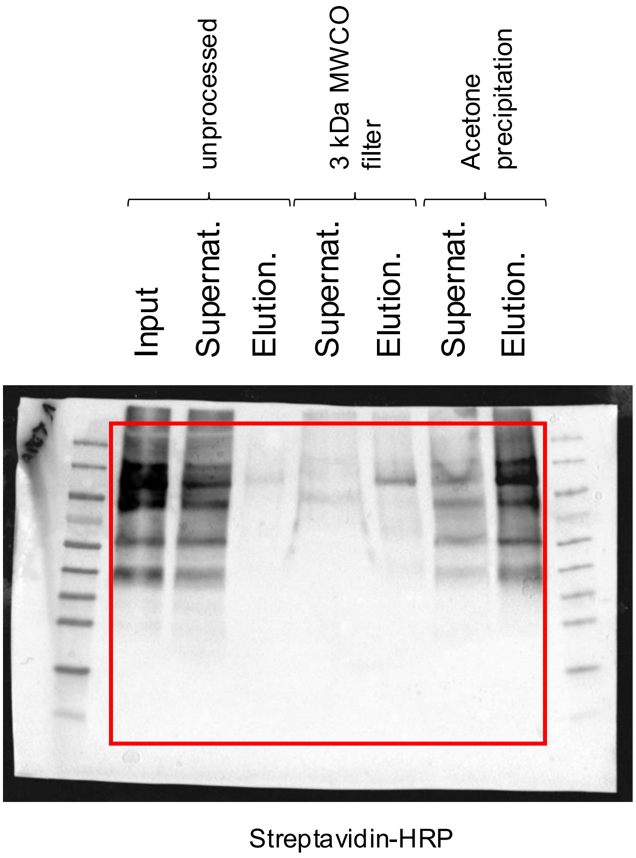

Figure S5A

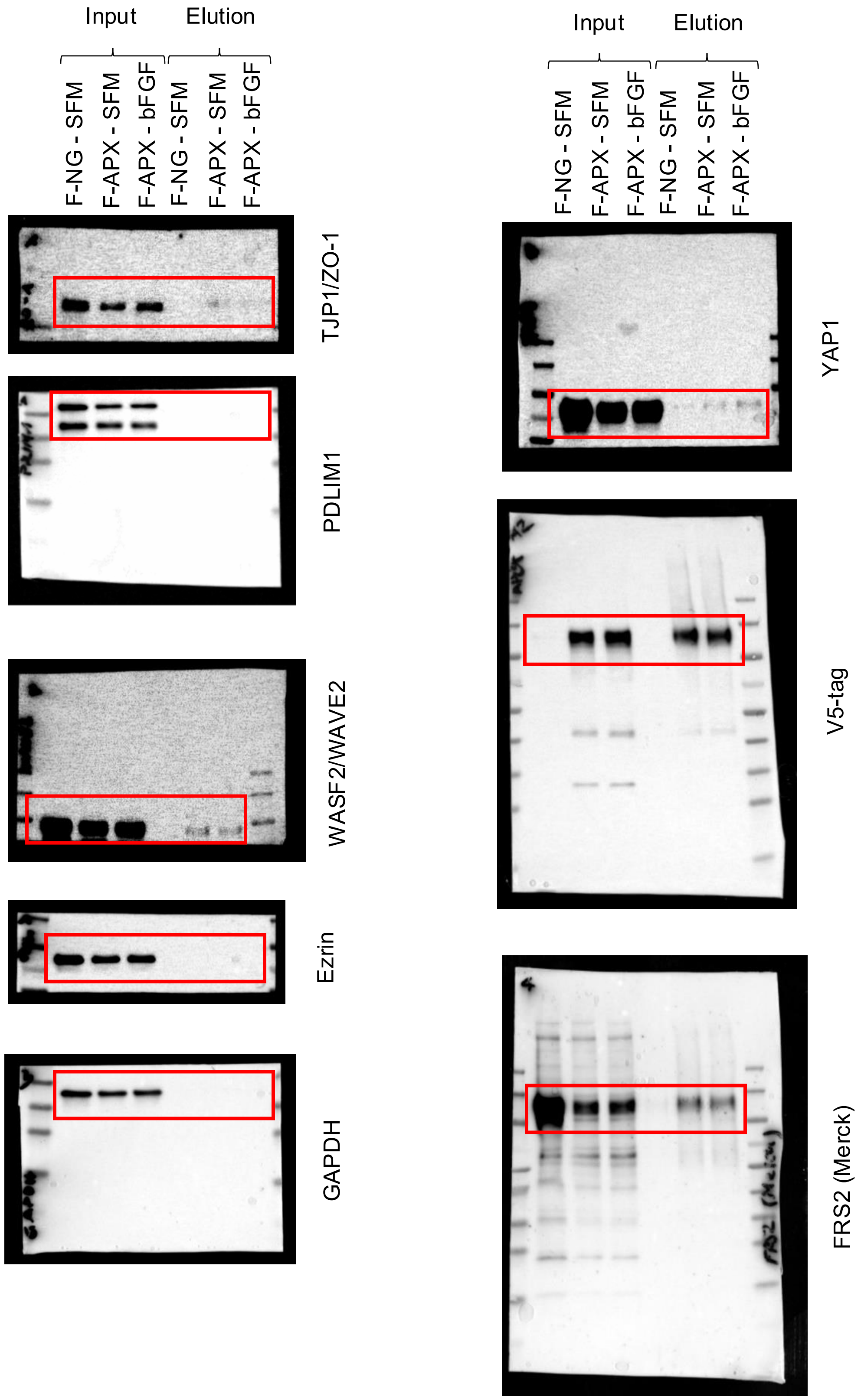

Input
Elution
F-NG - SFM
F-APX - SFM
F-APX - bFGF
F-NG - SFM
F-APX - SFM
F-APX - bFGF
TJP1/ZO-1
PDLIM1
WASF2/WAVE2
Ezrin
GAPDH
Input
Elution
F-NG - SFM
F-APX - SFM
F-APX - bFGF
F-NG - SFM
F-APX - SFM
F-APX - bFGF
YAP1
V5-tag
FRS2 (Merck)

Figure S6C

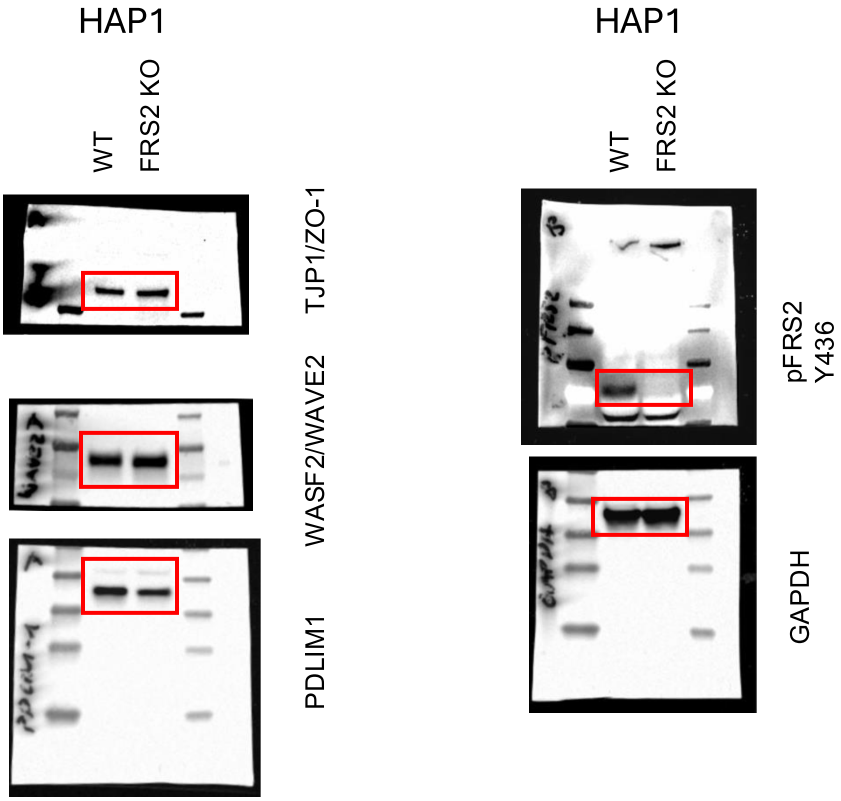
HAP1
WT
FRS2 KO
TJP1/ZO-1
WASF2/WAVE2
PDLIM1
HAP1
WT
FRS2 KO
pFRS2
Y436
GAPDH
